# Trypanosomiasis: An emerging disease in Alpine swift (*Tachymarptis melba*) nestlings in Switzerland?^☆^

**DOI:** 10.1016/j.ijppaw.2023.100895

**Published:** 2023-12-12

**Authors:** P. Cigler, G. Moré, P. Bize, C.M. Meier, C.F. Frey, W. Basso, S. Keller

**Affiliations:** a Institute for Fish and Wildlife Health, University of Bern, Länggassstrasse 122, 3012 Bern, Switzerland; b Institute of Parasitology, University of Bern, Länggassstrasse 122, 3012 Bern, Switzerland; c Swiss Ornithological Institute, Seerose 1, 6204 Sempach, Switzerland

**Keywords:** Avian trypanosomiasis, Alpine swifts, *Tachymarptis melba*, Trypomastigote, Amastigote-like form

## Abstract

Alpine swifts (*Tachymarptis melba*) are sub-Saharan migratory birds, which, in Switzerland, nest in colonies that have been continuously monitored for over 40 years. In the summer of 2022, despite favourable environmental conditions, an unexpectedly high number of sudden mortalities (30–80%) occurred in 20 to 45-day-old nestlings from several nesting sites, of which 3 were monitored in detail. Nestlings submitted for post-mortem analysis (n = 5) were in good body condition but exhibited extensive subcutaneous haematomas (n = 5), myocardial petechiae (n = 2) and stunted growth of primary feathers (n = 1). In all birds, 4–5 μm large, amastigote-like protozoans were identified in skeletal and cardiac muscle sections. These tissues tested positive in a PCR targeting the 18S-rRNA gene of *Trypanosoma* spp. Amplified sequences showed 99.63% identity with sequences of *Trypanosoma corvi* (JN006854 and AY461665) and *Trypanosoma* sp. (AJ620557, JN006841). 72 blood smears of 45-day-old nestlings from two colonies were assessed, of which 20 contained trypomastigote forms, some with high parasitaemia (highest average of 56.4 in 10 high power fields, 400x magnification). Trypomastigote morphometrics (n = 36; mean total length = 30.0 μm; length of free flagellum = 5.8 μm) were consistent with those of *T. bouffardi*. These findings suggest that an avian trypanosomiasis causing mass nestling mortality could be an emerging disease in Swiss Alpine swift populations.

## Highlights

• High Alpine swift nestling mortalities were observed across numerous Swiss colonies.
• Amastigote-like forms were observed in skeletal muscle, cardiac and adipose tissues.
• High trypomastigote burdens were identified on blood smears.
• Emerging avian trypanosomiasis has been identified as the most likely cause of mortality.

## Graphical abstract

**Figure:**
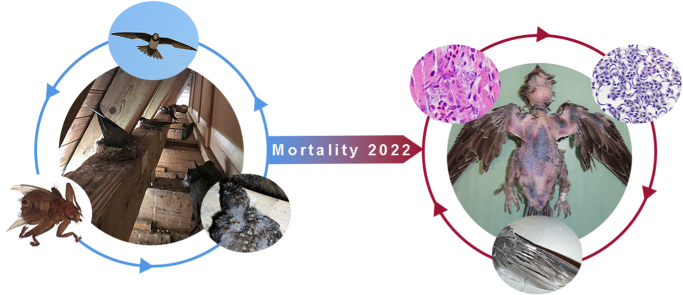

## 1 Introduction

Alpine swifts (*Tachymarptis melba,* formerly *Apus melba*) are Eurasian migratory birds that, in Europe, breed mainly around the Mediterranean basin to southern Germany, and migrate to sub-Saharan Africa in winter (Meier et al., 2020). Though usually nesting in steep cliffs, some colonies are situated in urban areas allowing for easier access and monitoring. In Switzerland this monitoring has been organised by the Swiss Ornithological Institute for over 40 years, offering unique insight into population dynamics, migratory paths, and health trends; information that is rarely available, but invaluable, when working with wildlife (Bize et al., 2003; Ilahiane et al., 2023; Meier et al., 2020). Though listed as Least Concern by the IUCN Red List (IUCN, 2016), the Alpine swift is considered a ‘priority species' for recovery programs in Switzerland and as such has benefited from numerous citizen conservation efforts, with special focus on the establishment and management of nesting sites (BirdLife, 2016).

Alpine swifts raise a brood of one to three nestlings each year which are fed by both parents from hatching until fledging at about 55 days of age (Bize et al., 2003). The monitoring of the Swiss population of Alpine swifts has established both fledging rates and population size trends throughout the years, indicating that, prior to 2022, cold and rainy weather conditions resulting in poor food availability (swifts feed exclusively on aerial insects) appeared to be the main influencing factor on fledging success (Arn-Willi, 1960). The only commonly reported parasites of Alpine swifts found throughout all Swiss colonies are Hippoboscid flies, specifically louse flies of the genus *Crataerina,* mainly *C. melbae* (Bize et al., 2003; Oboňa et al., 2019). This larviparous blood-sucking ectoparasite lays larvae that pupate immediately, overwinter in crevices around the nests, and hatch as adult louse flies in large numbers at the start of the breeding season to re-infest both adult and nestling swifts. This ectoparasite can be extremely abundant, infesting most nests with prevalence of >90% (Bize et al., 2003; Tella et al., 1998). Up to 100 flies have been recorded on individual nestlings (mean ± SE louse flies per nestling from hatching to fledging is 7.9 ± 0.1; n = 14,695 observations). Experimental manipulation of louse fly loads showed that they had only minor effects on the overall growth and weight of the birds (Bize et al., 2003, 2004).

Haemoparasites are common in wild birds (Jones, 2015; Valkiūnas et al., 2005). Depending on the infected avian species, age and immunological status of the bird, some haemoparasite species, for example *Plasmodium relictum, P. matutinum, P. homocircunflexum*, *Leucocytozoon simondi, Haemoproteus columbae,* and *H. mansoni,* can cause severe disease with potentially fatal outcome, while others (like *Trypanosoma* spp.) present low or no clinical significance (Atkinson, 2008; Bennett et al., 1994; Ilgūnas et al., 2016; Meister et al., 2021, 2023; Valkiūnas et al., 2005).

Avian trypanosomes can be found in a large array of bird species all over the world, excluding only the north and south poles (Zídková et al., 2012) Unlike other avian hemoparasites, avian trypanosomes develop a low parasitaemia, making them less common when evaluating blood smears (Molyneux, 1973; Bennett et al., 1994).

Trypanosomes are unicellular, flagellated protozoan parasites in the order Trypanosomatida that can infect humans and animals. This order includes important species within the genera *Leishmania* and *Trypanosoma*, known to cause severe disease and economic loss (Lukeš et al., 2018). Information available on avian trypanosomes is scarce in comparison to that on species affecting mammals, such as *Trypanosoma cruzi* causing Chagas disease, *T. brucei*, *T. congolense* and *T. vivax*, causing Nagana in Africa, *T. equiperdum* responsible for dourine and *T. evansi* causing surra (Deplazes et al., 2016; Cayla et al., 2019).

In the past, almost 100 avian *Trypanosoma* species were described, mainly based on the host in which they were observed, assuming host specificity. However, with advances in a combination of morphometrics, molecular and phylogenetic studies, only a few species remain valid today (Zídková et al., 2012). Presently, avian trypanosomes are divided in at least 14 different lineages (I-XIV) and arranged into three major groups (i.e., Group A: *T. bennetti*, Group B: *T. corvi/T. culicavium* and Group C: *T. avium/T. thomasbancrofti*) (Santolíková et al., 2022; Zídková et al., 2012). Of these species, several have been detected in different avian hosts with generally no clinical relevance. Only *T. bouffardi* has been documented to cause high parasitaemia in free-ranging African passerine birds (Molyneux, 1973) and in experimentally infected canaries (Molyneux et al., 1983). Despite high parasite burdens, no clinical symptoms were reported in free ranging birds (Molyneux, 1973). However, this species has been only described by morphological studies (Bennett et al., 1994) with no molecular data available. Some of the species or lineages have been shown to have haematophagous insects as vectors, for example: hippoboscid flies for *T. corvi*, culicine mosquitoes for *T. culicavium* and blackflies for *T. avium* (Santolíková et al., 2022; Zídková et al., 2012).

Despite the well described co-existence of numerous swift species with their species-specific louse flies, Alpine swifts, as well as other members of the swift family (Apodidae) were rarely documented to carry haemoparasites (Ilahiane et al., 2023). A study conducted shortly prior to the present report, did not detect haemosporidian parasites in Swiss Alpine swifts and its louse flies (Ilahiane et al., 2023). Regarding the prevalence of avian trypanosomes in hippoboscid flies, a study from 2022 did not find any in the common-swift (*Apus apus*) specific hippoboscid fly *Crataerina pallida* (Santolíková et al., 2022).

In the summer of 2022 sudden, unexpectedly high Alpine swift nestling mortalities began to be reported across multiple monitored Swiss colonies, as well as an increase in unusual growth defects of their primary feathers and an increase in fledglings being presented to rescue centres with flight feather deformities. Despite overall favourable conditions when compared to previous years with poor fledging rates, mortalities ranged from an average of 30–50% per colony, with peaks reaching 80% of all nestlings. No morbidities or mortalities were documented amongst adult birds, and the behaviour of the nestlings remained mostly unchanged up until death.

This study aimed to investigate the potential causes for the mortalities using a combination of histological, parasitological, haematological, and molecular methods.

## 2 Materials & methods

### 2.1 Material collection

**Fig. 1:**
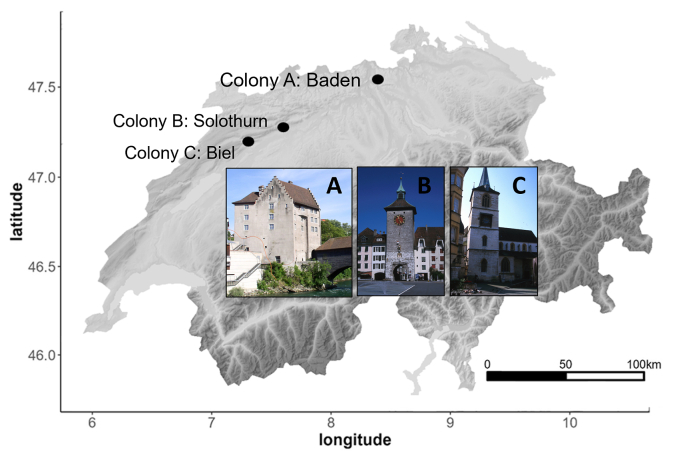
Map of Switzerland with the locations and appearance of the three evaluated colonies (A, B, C).

In 2022, seven urban Alpine swift colonies were routinely monitored (every 3–15 days) throughout the breeding season by the Swiss Ornithological Institute to record egg laying, hatching and fledging success. Three colonies were included in this study. Colony A is found in the *Landvogteischloss* in Baden (2′665′757 N, 1′258′394 E), Colony B is located at the *Bieltor* in Solothurn (2′607′277 N, 1′228′580 E) and colony C at the *Stadtkirche* Biel (2′585′433 N, 1′221′176 E) (Fig. 1).

**Fig. 2:**
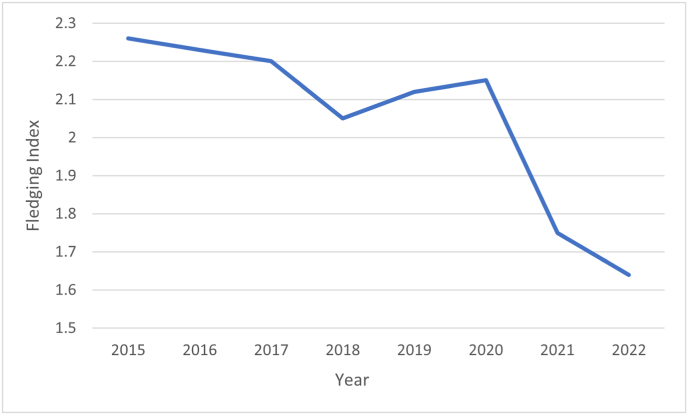
Fledging indexes of 7 Swiss Alpine swift colonies from 2015 to 2022.

Mean fledging rates from 2015 to 2022, averaging the successful number of nestlings fledged per nest, were evaluated for all seven colonies (Fig. 2). 2021 had exceptionally low fledging success explained by poor weather conditions, while 2022 should have been a good fledging index year, based on favourable weather conditions.

Colonies B and C were evaluated in more detail. The nestlings were individually ringed and measured as described in Bize et al., (2006) (body mass to the nearest 0.1 g, wing length to the nearest mm, and sternum length to the nearest 0.1 mm), and any abnormalities, including pale coloration of the feet, bruising or flight feather deformities, were documented. The outcome of each nestling was assigned as either fledged, deceased or unaccounted for but presumed dead. In 2022, 99 nestlings were ringed in colony A, 74 nestlings in colony B and 95 in colony C.

Additionally, 72 blood smears of 45-day-old nestlings from colonies B and C, collected for other research purpose by the Swiss Ornithological Institute, were obtained. Blood was collected from the ulnar vein using a Microvette© CB 300 lithium-heparin (Sarstedt AG, Germany) and 10 μl of whole blood was used to make thin smear. The smear was air dried before being stained within one week after blood collection with a fast-acting variation of May-Grünwald Giemsa staining (kit RAL 555, VWR, ref 720-0351). All animals were sampled in accordance with national legislation following Permit Number LU01/2022 and National Animal Experimentation Permit Number 34497 for the purpose of a separate long-term study carried out by the Swiss Ornithological Institute.

### 2.2 Post-mortem examination

Five 30-45-day-old, deceased nestlings from three colonies (three from colony A, one from colony B, one from colony C) were submitted to the Institute of Fish and Wildlife health (FIWI) for post-mortem examination between July and August 2022.

All animals underwent full post-mortem examinations; however, due to the state of decay, two from colony A were unsuitable for histopathology. Brain, heart, lung, liver, kidney, skin, and skeletal musculature were sampled from the remaining three birds. The spleen and bursa fabricii were sampled in two birds. All samples were fixed in 10% buffered formalin, processed, embedded in paraffin, and subsequently trimmed and stained with H&E according to standard protocols of the Institute of Animal Pathology of the University of Bern, Switzerland. Additionally, samples of brain, liver, lung, spleen, and kidney were stored at −20 °C for ancillary testing.

#### 2.2.1 Ancillary diagnostics

Brain and liver tissues from all five birds were tested in-house for the presence of Usutu virus (Flavivirus) using a reverse transcription (RT) PCR proceeding according to Pisano et al. (in preparation). The same samples were tested using West-Nile Virus (Flavivirus) qPCR adapted from Eiden et al. (2010).

Skin, muscle, and liver samples of two nestlings (Colony B and C) were submitted to Laboklin GMBH & CO.KG (97688 Bad Kissingen, Deutschland) for Polyomavirus (Avipolyomavirus) and Beak and feather disease virus (Circovirus) detection using real-time PCR.

Skin, lung, and spleen samples from one nestling (Colony B) and liver, lung and spleen from a second nestling (Colony C) were submitted to the Institute of Bacteriology, University of Bern, Switzerland, for anaerobic, *Salmonella* spp. and *Yersinia* spp. cultures. BD™ Brucella Blood Agar with Hemin and Vitamin K1 (Becton Dickinson GmbH, Heidelberg, Germany), Brilliance™ Salmonella (Oxoid Limited, United Kingdom) and Yersinia Selective Agar (Oxoid Limited, United Kingdom) were used respectively.

### **2.3** Kinetoplastid protozoan PCRs

Tissue samples from three animals (one from each colony) were individually subjected to testing as tissue pools (liver, spleen, and skeletal muscle) for DNA extraction using a commercial kit according to manufacturer instructions (Blood and Tissue kit QIAGEN, Germany). The DNA samples were first processed by PCR targeting a fragment of the Internal Transcribed Spacer 1 (ITS1) from *Leishmania* spp., as previously described by Müller et al. (2009).

Subsequently, a PCR targeting the 18S rRNA gene-fragment of *Trypanosoma* spp., using only the internal primers described by Noyes et al. (1999), was performed. This PCR was conducted using QIAGEN Multiplex Mix 2× in a final volume of 25 μl and using 2.5 μl of each DNA sample. The following cycling program was used: 95 °C for 15min, 35 cycles (94 °C for 30sec, 55 °C for 60sec, 72 °C for 30sec), final extension of 72 °C for 10min. Each run contained a positive control and a non-template control (NTC). The amplification products of both PCRs were electrophoresed in 1.5% agarose gel wells stained with ethidium bromide and observed and photographed (not shown) in a UV light image system (E-Box, Vilber, France). The resulting PCR amplicons were purified using a commercial kit (DNA Clean & Concentrator-5, Zymo Research, Irvine, USA) following manufacturer instructions and submitted for Sanger sequencing to Microsynth, Balgach, Switzerland (https://srvweb.microsynth.ch/) with the two primers used for each amplification. Sequences were aligned and analysed using the Geneious Prime software (https://www.geneious.com). The resulting consensus sequences were compared with those available in GenBank by nucleotide BLAST analysis (http://blast.ncbi.nlm.nih.gov/Blast.cgi).

### 2.4 Evaluation of blood smears

All blood smears collected in 2022 (n = 72) were air dried, stained using a fast-acting variation of May-Grünwald Giemsa commercial kit (RAL 555, VWR, ref 720-0351) and microscopically investigated at the FIWI by one examiner for the presence or absence of trypomastigotes. When trypomastigotes were detected (n = 20 samples, here forth termed ‘positive animals’), the number of parasites, thrombocytes, mononuclear cells, and granulocytes was estimated by counting 10 randomly selected High Power Fields (HPF). This was also performed in 20 randomly selected samples, in which no trypomastigotes were detected (termed ‘negative animals’). Due to the inconsistent quality of the smears, including varying thickness and cell distribution, cell lysis and inconsistencies in staining, leukocytes were not further differentiated. Additionally, indication of polychromasia was noted in each smear based on chromatin structure, colour of the cytoplasm and size of the nucleus.

#### 2.4.1 *Trypanosoma* sp. morphometrics

Trypomastigotes (between 6 and 15 per sample) found in three samples from 2022 were measured. The smears were evaluated using a Nikon Eclipse *Ci* microscope and photographed with a calibrated Nikon Camera (model DFK 23UP031). Measurements were taken with the NIS-Elements D software (Version 5.02.03 64 bit) and included: total length without flagellum (PA), posterior end to centre of kinetoplast (PK), posterior end to centre of nucleus (PN), centre of nucleus to anterior end (NA), centre of kinetoplast to centre of nucleus (KN), body width at the centre of the nucleus (BW), length of free flagellum (FF), area of the trypomastigote (AT) and area of the nucleus (AN) (Bennett et al., 1994).

## 3 Results

### 3.1 Field observations

**Fig. 3:**
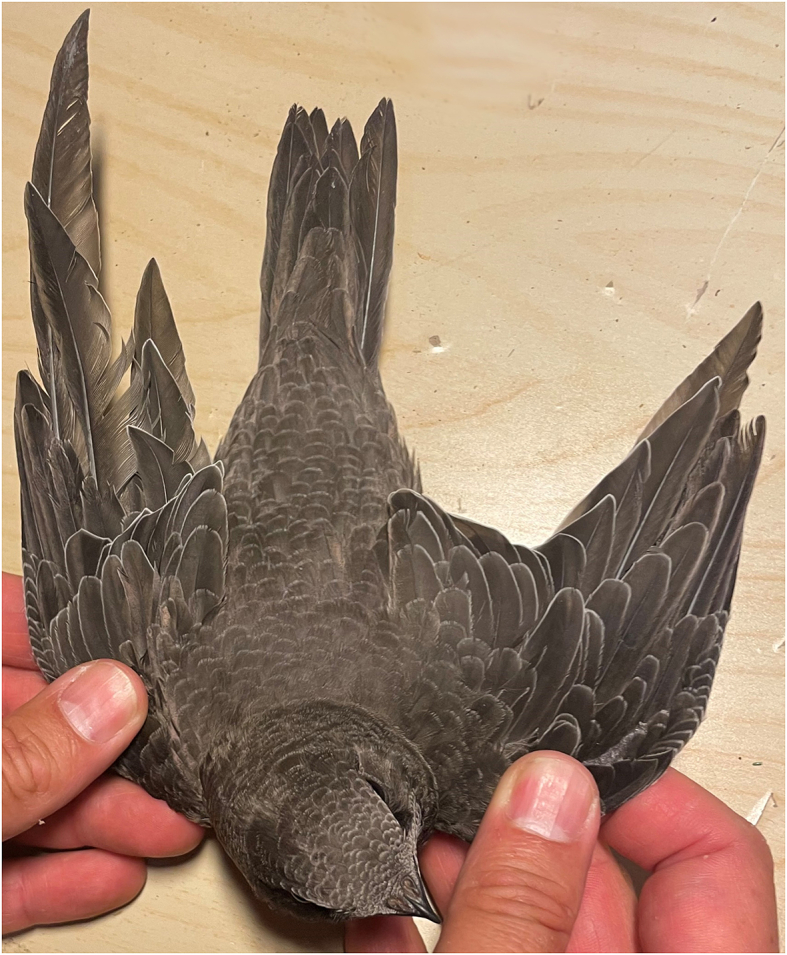
Missing (left wing) and poor quality (right wing) primary feathers on a 45-day-old nestling.

The affected nestlings showed pale coloration of mucous membranes and feet, alongside moderate to severe bruising across the body. These signs were observed earliest in nestlings aged 14–20 days, although most observations were in nestlings aged 20–45 days. Other changes in the form of primary feather abnormalities, including stunted growth, retained feather shafts and/or blood keels, were observed in nestlings aged 40 days and over (Fig. 3). However, as these changes were not noted consistently, the exact numbers are unknown.

A considerable louse fly burden was described on both healthy and affected nestlings, with a mean of 5.5 ± 0.2 per nestling throughout development (690 observations). The highest burden on an individual nestling observed in 2022 was 39 louse flies.

The numbers of fledged, deceased or missing (but presumed dead) nestlings, as well as the average age and weight at the time of death were evaluated for colonies A, B and C and compiled in Table 1.

**Table 1:** Summary of the number of nestlings ringed, fledged and deceased or missing in colonies A, B and C, including median, minimum, and maximum age and weight at death. Ref. * = median (min/max).

	Colony A	Colony B	Colony C
Nr. of ringed nestlings	99	74	95
Nr. of fledged nestlings	49	54	26
Nr. of deceased or missing	50	20	69
Age of death (days)*	n/a	21 (14/49)	24 (14/49)
Weight at death (g)*	n/a	83.7 (57.7/107.7)	88.6 (46.8/112.2)
Mortality (%)	50.5	27.0	72.6

### 3.2 Post-mortem examination and histopathology

**Fig. 4:**
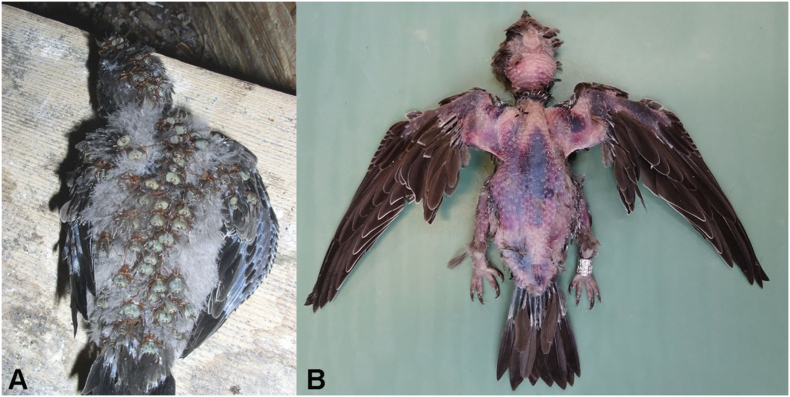
Distribution of louse flies on an Alpine swift nestling (A) compared with the distribution of bruising on post-mortem examination with plumage removed (B).

All necropsied nestlings (n = 5) showed identical findings on gross pathology, including extensive subcutaneous haemorrhages and pale discoloration of mucous membranes and feet. The haemorrhages were randomly distributed; however, were more prominent along the back, the top of the head, both sides of the wings and on the breast (Fig. 4). In animals sampled within 24 h of death, myocardial petechiae could be observed (n = 2). Significant splenomegaly was present in all animals. All birds were in good body condition, had considerable fat reserves and full intestinal tracts. One bird had significantly shorter primary feathers on one wing, without indication of a trauma. The remainder of the plumage and the uropygial gland were unremarkable.

**Fig. 5:**
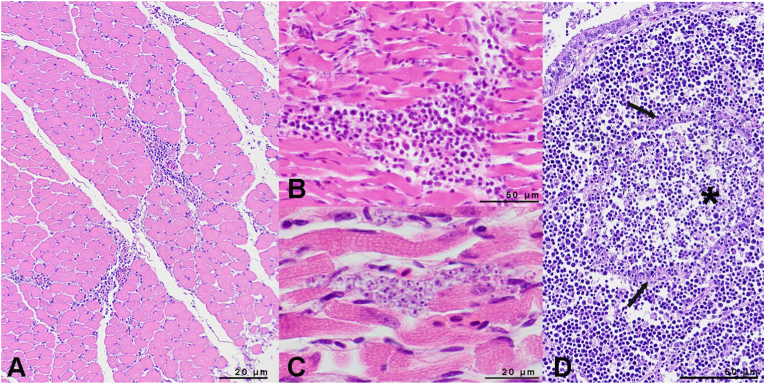
Skeletal musculature of a nestling Alpine swift showing infiltrations of mononuclear inflammatory cells (A, B) and presumably extracellular, amastigote-like structures (C). Bursa fabricii of a nestling Alpine swift with depletion of the medullary follicle with lymphocytolysis (asterisk) and a distinct epithelium (arrows) (D).

Histopathology (n = 3, one from each colony) confirmed extensive areas of haemorrhage in subcutaneous tissue, reaching into the underlying musculature. The epidermis and feather follicles were without abnormality. Pectoral and wing (mainly biceps) musculature showed multifocal, interstitial, often perivascular accentuated small to medium sized infiltrations of mononuclear inflammatory cells (macrophages, lymphocytes and plasma cells, Fig. 5A/B). In two birds, multifocal haemorrhaging was observed along the epicardium and, in part, infiltrating into the underlying myocardium. Sporadically distributed throughout the myocardium, a mixed inflammation composed of macrophages, lymphocytes, plasma cells and few heterophils, was seen in all three birds.

In proximity to the inflammatory foci, 4–5 μm, round-to-oval structures containing two basophilic nuclear-like structures were observed. These structures resembled the amastigote stages of kinetoplastid parasites and were found primarily in skeletal and cardiac musculature, as well as sporadically dispersed amongst fat cells in connective tissue and appeared to be extracellular (Fig. 5C). No trypomastigotes could be identified within blood vessels.

The bursa fabricii examined in two birds demonstrated a depletion of the medullary follicles characterized by moderate to severe lymphocytolysis, surrounded by a distinct epithelium (Fig. 5D). The spleens were difficult to assess due to autolytic changes. The lymphoid follicles were hypocellular and could not be clearly discriminated from the perivascular sheets, indicative of a mild lymphoid depletion.

All other organs were generally unremarkable with no indications of any other underlying diseases.

### 3.3 Ancillary diagnostics

All birds (n = 5) tested negative for both West Nile and Usutu viruses. Both birds examined for Polyoma- and Circovirus tested negative.

A bacteriological examination of liver, lung, and spleen in one nestling resulted in growth of unspecific flora interpreted as post-mortem overgrowth.

### 3.4 Molecular diagnostics

The initial *Leishmania* spp. PCR ITS1 resulted positive in all three samples with an approximate 350 bp length band, and thus larger than expected for *Leishmania* spp. Sequencing analysis from one of these samples (consensus sequence of 260 bp) showed 97–100% identity with several *Trypanosoma* spp. 18S rRNA sequences, but with a low coverage (14–16%).

Tissue pools of the nestlings (3/3) were positive for *Trypanosoma* spp. based upon results from the 18SrRNA PCR (gel not shown).The amplicon from one nestling was sequenced and the consensus sequence of 539 bp (primers trimmed GenBank accession no. OR598759) which showed an identity of 99.63% with 100% coverage with several sequences: *Trypanosoma corvi* (JN006854 and AY461665, from *Buteo* from Czechia and *Corvus frugilegus* from the United Kingdom, respectively), *Trypanosoma* sp. (LZ 2011, JN006841, from *Ficedula albicollis* in Czechia) and *Trypanosoma* sp. AAT (AJ620557, from *Strepera* sp., an Australian bird). Our sequence showed two mismatches with all the referenced sequences.

### 3.5 Evaluation of blood smears

Of the 72 evaluated blood smears from the 2022 season (colony B and C), 65 were from nestlings which fledged at the end of the season (90.2%). Overall, 27.8% of samples contained trypomastigotes (20/72). Seven animals died after 45 days-of-age, of which six (85.7%) had trypomastigotes and one did not (14.3%). Of the nestlings that fledged (n = 65), 14 had trypomastigotes (21.5%) visible on blood smear.

**Fig. 6:**
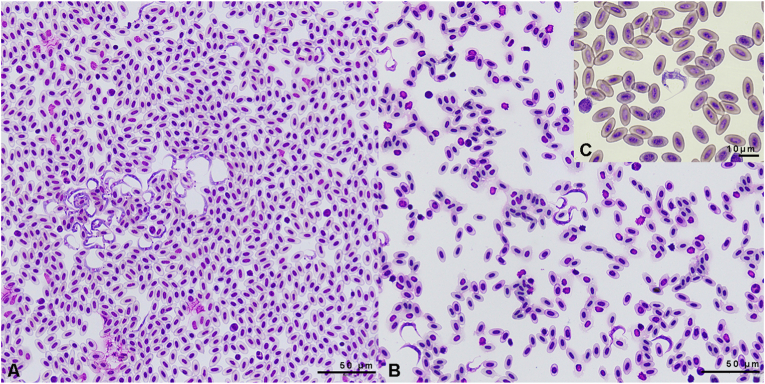
Blood smears of nestling Alpine swifts with high (A) and moderate (B) trypomastigote burdens. Close-up of a trypomastigote between erythrocytes (C).

Forty blood smears were examined in detail. An average trypomastigote burden of 7.9 trypomastigotes per 10 HPF was observed amongst positive animals (n = 20). In the sample with the highest burden, 56.4 trypomastigotes per 10 HPF were found. In most smears, the trypomastigotes were observed in clusters of rosette-like formations (Fig. 6).

No clear difference was observed in the thrombocyte counts between positive and negative nestlings, with an average of 13.9 thrombocytes per 10 HPF.

**Fig. 7:**
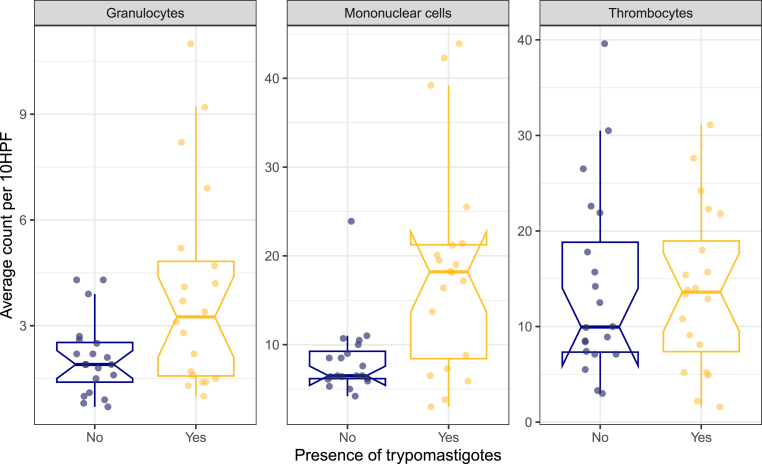
Box plots comparing average counts per 10 HPF of granulocytes, mononuclear cells, and thrombocytes between positive (n = 20) and negative (n = 20) 45-day-old Alpine swift nestlings sampled in 2022.

Positive nestlings showed a higher level of mononuclear cells (18.6 per 10 HPF) in comparison with negative ones (8.2 per 10 HPF). Positive nestlings also showed a slightly higher level of granulocytes (3.9 per 10 HPF) when compared to negative ones (2.1 per 10 HPF). All counts, including standard deviation (SD), are compiled in Table 2 and Fig. 7.

**Table 2:** Average counts of 10 high-power-field counts (HPF) 400× magnification of positive and negative nestlings, with Standard deviation and range in brackets.

Per 10HPF	Trypomastigotes	Thrombocytes	Mononuclear Cells	Granulocytes
Average positive n = 20	7.9 (13.2; 0.1–56.4)	13.9 (8.2; 1.6–31.1)	18.6 (11.7; 3.0–43.9)	3.9 (2.8; 1.0–11.0)
Average negative n = 20	0	14.0 (9.5; 3.0–39.6)	8.2 (4.1; 4.2–23.9)	2.1 (1.0; 0.7–4.3)

### 3.6 Trypomastigote morphometrics

Trypomastigote morphometrics were similar in all evaluated blood smears (Fig. 6C). The total length without the flagellum was on average 30.0 μm, based on n = 36 from three blood smears, compiled in Table 3.

**Table 3:** Average morphometric measurements of 36 trypomastigotes observed in 2022 from three 45-day-old Alpine swift nestlings. SD: Standard deviation.

Measurement	Average *μm* (SD; Min-Max)
**PA:** total length without flagellum	30.0 (2.3; 25–35)
**PK:** posterior end to centre of kinetoplast	4.7 (2.2; 1,5–10.2)
**PN:** posterior end to canter of nucleus	13.6 (1.8; 10.4–16.7)
**NA:** centre of nucleus to anterior end	15.7 (1.6; 12–18)
**KN:** centre of kinetoplast to centre of nucleus	9.4 (0.8; 7.3–11.5)
**BW:** body width at the centre of the nucleus	3.1 (0.4; 2.3–4.2)
**FF**: length of free flagellum	5.8 (0.9; 4.4–7.7)
**AT:** area of the trypomastigote	56.7 (5.7; 46.3–66)
**AN:** area of the nucleus	4.2 (0.9; 2.7–6.2)

## 4 Discussion

Initial results evaluating the 2022 Alpine swift nestling mortalities observed throughout Switzerland suggest the emergence of a potentially novel infection caused by a pathogenic avian *Trypanosoma* species.

The main clinical signs observed in the field were generalized pallor and extensive bruising, resulting from severe subcutaneous haemorrhages. We hypothesise that louse fly bites trigger said haemorrhages, as the distribution of bruising and infestation sites by this blood sucking ectoparasite correspond. Based on these findings, death is suspected to be caused by a severe anaemia and hypovolemia following the exorbitant haemorrhaging. Having excluded the possibilities of trauma (e.g., falling out of a nest, aggression through con- or allospecifics), it is believed that the extensive blood loss is the result of a coagulopathy, of which the pathophysiology mechanisms are still unknown. Given that high louse fly burdens have been historically well documented on Alpine swift chicks without clinical signs, significant effect on development or mortalities (Bize et al., 2003, 2004, 2006), it is believed that the trypanosomes play a key role in the development of this disease.

No trypomastigotes were observed within vessels on histopathology. Instead, amastigote-like structures were seen in all examined birds within skeletal and cardiac musculature. The exact characterization, including whether they are located inter- or intracellularly, and clinical relevance remains unclear. An intracellular amastigote form has been described in *T. cruzi* and *Leishmania* spp. and represents a persistent form of infection in the host (De Souza et al., 2010; Kima, 2007). The *T. cruzi* amastigotes can invade and replicate within different cells including, but not limited to those of heart musculature, skeletal musculature, gastrointestinal tract, pancreas, spleen, lung and adipose tissue (Guarner et al., 2001). A potentially dividing amastigote form was also reported in lymphatic tissue of a *T. microti*-infected short-tailed field vole (*Microtus agrestis*) (Molyneux, 1969). It is possible that the amastigote-like form serves a similar function in the Alpine swift; however, why they appear to be located extracellularly remains unclear. Extravascular migration of trypomastigotes and accumulation in interstitial spaces of striated muscle or adipose tissues may be associated with an impending morphological change to the amastigote-like stage. Extracellular localization of trypanosomes in several tissues has been observed in *T. brucei* (Crilly and Mugnier, 2021). The only descriptions of a similar amastigote-like structure in a bird were documented in blood smears of a *T. bouffardi* infection (Molyneux and Robertson, 1974; Bennett et al., 1994), as well as in bone marrow of *T. corvi* infected Rufous treepies (*Dendrocitta vagabunda*) (Nandi and Bennett, 1994). Nevertheless, as all examined tissues were sampled from deceased birds, the potential that the observed structures represent moribund trypanosomes requires further investigation (Nandi and Bennett, 1994).

Overall, these findings are significant, as avian trypanosomes are generally considered apathogenic, incidental findings, resulting in a low parasitaemia with no concurrent clinical signs (Bennett et al., 1994; Santolíková et al., 2022). The only avian trypanosome which fulfilled Koch's postulates and was described to be pathogenic, with a higher parasitaemia when compared to other species (*T. everetti*, *T. corvi*, among others) is *T. bouffardi* (Molyneux, 1973). Experimentally infected canaries developed lesions of the heart and spleen, similar to those seen in Alpine swifts, including mononuclear infiltrations of the myocardium and splenomegaly (Molyneux et al., 1983).

Morphometrics of trypomastigotes observed in Alpine swifts were also similar (but not identical) to those reported for *T. bouffardi* (Molyneux and Robertson, 1974). Amplicon sequencing revealed a 99.6% identity with sequences of *T. corvi* (JN006854 and AY461665) and two unnamed *Trypanosoma* spp. (JN006841, from *Ficedula albicollis;* and AJ620557, from *Strepera* sp.). All these sequences belonged to the genetic group B (*T. corvi/T. culicavium*), containing several lineages or species (Santolíková et al., 2022; Zídková et al., 2012). *Trypanosoma corvi* trypomastigotes, even so-called ‘slender’ forms, are considerably larger than those seen in the Alpine swift (Molyneux and Gordon, 1975; Pornpanom et al., 2019; Nandi and Bennett, 1994). Further still, the posterior end to kinetoplast (PK) and centre of nucleus to anterior end (NA) measurements are smaller than described in *T. corvi* (Nandi and Bennett, 1994). This species has therefore been *a priori* ruled out. Since research on *T. bouffardi* was carried from the 1970s until the early 90s (Bennett et al., 1994; Molyneux and Robertson, 1974; Molyneux et al., 1983), no genetic data is available to support or disprove the hypothesis that the same species is affecting Alpine swifts. For this reason, the species is referred to as *T. bouffardi*-like in the remainder of the discussion.

As trypanosomes require a vector to spread, it is believed that the louse flies found in the Alpine swift colonies (*Crataerina melbae*) represent potential competent vectors. Field observations suggest that louse flies initially prefer to parasitize on feathered birds, e.g., adults at the beginning of the season, which are the most likely source of infection, despite not showing clinical signs. After being ingested through the blood meal, the trypanosomes likely replicate in the gut of the louse flies. Birds may become infected following ingestion of louse flies, as is described in other members of the trypanosome genetic group B, e.g. *T. corvi* (Baker, 1956; Votýpka et al., 2004), or by potential contamination of skin (and microlesions, i.e. louse fly bites) or mucous membranes with louse fly faeces containing trypanosomes, as described for the vector transmission of some avian trypanosomes, as well as *T. cruzi* (Baker, 1956; Fialová et al., 2021; Deplazes et al., 2016). True vector competency, however, has yet to be confirmed, as active replication of the *T. bouffardi*-like parasites in the gut or other tissues of the louse flies has not been demonstrated.

The origin of the novel trypanosomiasis is similarly uncertain. If the suspicion that the parasite is *T. bouffardi* is correct, it most likely originated from Africa. Though reported to be present in wild songbird populations in Botswana (Molyneux, 1973) and sub-Saharan region (Bennett et al., 1994), no up-to-date information exists of the potential spread and prevalence in other parts of Africa, and/or other continents. Biologging data of the Alpine swifts shows that some birds overwinter in areas which could potentially overlap with the natural range of this haemoparasite (Meier et al., 2020), but the question of how the swifts may have gotten into contact with a songbird-specific vector, given they generally do not land in their overwintering period (Liechti et al., 2013), remains open. As swifts are strict aerial insectivores per-oral infection with consumption of an infected, free-flying vector in Africa, is suspected.

Reviewing the 2022 fledging and developmental data from two large, affected colonies, it can be assumed that the main onset of mortalities, most likely associated with the novel trypanosomiasis, occurred at around 20–30 days of age. As such, the blood smears collected from 45-day-old nestlings and examined in this study represent a biased sample set of nestlings that were older than the peak of mortalities (median 21/24 days – Colony B/C). As 14 of the 20 nestlings with trypanosomes on blood smears fledged successfully, it is assumed that these individuals were infected later and/or were able to withstand the infection, and thus cannot be used as a direct representation of the haematology of severely infected animals which succumbed to the disease.

Despite this, comparison of nestlings with trypomastigotes to those without revealed a higher mononuclear cell count, as well as a slightly higher level of granulocytes. Thrombocytes showed to have little difference between affected and unaffected nestlings. A degree of polychromasia was observed, with no difference between affected and unaffected nestlings. Given that no haematology reference values are available for Alpine swifts, or nestlings in the age group described in this study, it is difficult to conclude to which extent the polychromasia observed was pathological, and to which extent it was part of the natural degree observed in young, rapidly growing birds.

In general, these findings must be interpreted with care. The quality of the blood smears, and thus the distribution of trypomastigotes, leucocytes and thrombocytes varied highly between and within the smear, rendering them less reliable. In the future, other, more precise methods should be implemented to confirm the suspicion of a coagulopathy and/or immunosuppression.

Overall, there is a distinct paucity of knowledge on the effects of avian trypanosomes on their hosts, especially on the red blood cells (RBCs). However, within the mammalian African trypanosomiasis complex, anaemia represents the primary clinical sign, and has, as such, been closely studied (Musaya et al., 2015; Stijlemans et al., 2018).

Trypanosome infections in mammals may cause anaemia in a number of ways, including massive erythrophagocytosis by an activated mononuclear phagocytic system (MPS) of the host (Igbokwe and Nwosu, 1997), acute haemolysis due to parasite proliferation (Ekanem and Yusuf, 2008; Ogunsanmi and Taiwo, 2001; Saleh et al., 2009; Umar et al., 2007), and/or an increased susceptibility of RBC membranes to oxidative damage resulting from a depletion of glutathione on the surface of the RBCs (Akanji et al., 2009; Igbokwe et al., 1994, 1996; Taiwo et al., 2003). Evidence of an increased erythrophagocytosis, such as a splenic or hepatic hemosiderosis, could not be observed in the nestlings. The high trypomastigote burdens, and rosette-like formations seen on blood smears suggesting active proliferation could potentially result in acute haemolysis.

Like with RBC, no information is available on the effects of trypanosomiasis on white blood cell (WBC) counts in birds. In mammals, again specifically within the African complex, lower WBC counts, lymphocytes and neutrophiles were attributed to immunosuppression caused by the trypanosomes (Abubakar et al., 2005; Ekanem and Yusuf, 2008). The reason for the excessive depletion of the lymphatic tissues and higher mononuclear cell counts seen in trypanosome positive nestlings cannot be explained at the current state of research. Given that the post-mortem examinations and blood smears were from 30-to-45-day-old animals, thus older than those that died at the peak mortality period (median 21/24 days – Colony B/C), the immune system may have had time to react with an increase in circulating mononuclear cells. However, as the exact time-point of infection in the examined animals remains unknown, this speculation remains to be confirmed or disproved with future research investigating the maturation of the immune system in both infected and healthy Alpine swift nestlings.

## 5 Conclusion

Given the severity of the parasitaemia and haemorrhaging observed and having excluded other likely causes (ex. viral infections), we believe this to be the first description of a mass mortality event, and potential emerging disease, caused by an avian trypanosome. Due to trypomastigote morphometrics, the presence of the amastigote-like form, and the nature of the infection, but in the absence of molecular data for comparison, the species has been coined *T. bouffardi*-like. Further research is paramount to better understand the host-pathogen interactions at play, as well as potential underlying environmental conditions that may have propagated the severity of this outbreak.

## 6 Ethics declarations

All free-ranging animals were sampled by the Swiss Ornithological Institute in accordance with national legislation following Permit Number LU01/2022 and National Animal Experimentation Permit Number 34497. Post-mortem examinations were conducted at the Institute for Fish and Wildlife Health in the scopes of the national general wildlife health surveillance.

## Declaration of competing interest

The authors declare that they have no known competing financial interests or personal relationships that could have appeared to influence the work reported in this paper.
